# Localization, Shedding, Regulation and Function of Aminopeptidase N/CD13 on Fibroblast like Synoviocytes

**DOI:** 10.1371/journal.pone.0162008

**Published:** 2016-09-22

**Authors:** Rachel L. Morgan, Nilofar Behbahani-Nejad, Judith Endres, M. Asif Amin, Nick J. Lepore, Yuxuan Du, Andrew Urquhart, Kevin C. Chung, David A. Fox

**Affiliations:** 1 Division of Rheumatology and Clinical Autoimmunity Center of Excellence, Department of Internal Medicine University of Michigan, Ann Arbor, Michigan, United States of America; 2 Department of Orthopedic Surgery, University of Michigan, Ann Arbor, Michigan, United States of America; 3 Division of Plastic Surgery, Department of Surgery, University of Michigan, Ann Arbor, Michigan, United States of America; Universitatsklinikum Jena, GERMANY

## Abstract

Aminopeptidase N/CD13 is highly expressed by fibroblast like synoviocytes (FLS) and may play a role in rheumatoid arthritis (RA). CD13 was previously detected in human synovial fluid where it was significantly increased in RA compared to osteoarthritis. In this study we found that CD13 in biological fluids (plasma, synovial fluid, FLS culture supernatant) is present as both a soluble molecule and on extracellular vesicles, including exosomes, as assessed by differential ultracentrifugation and density gradient separation. Having determined CD13 could be released as a soluble molecule from FLS, we examined potential mechanisms by which CD13 might be shed from the FLS membrane. The use of protease inhibitors revealed that CD13 is cleaved from the FLS surface by metalloproteinases. siRNA treatment of FLS revealed one of those proteases to be MMP14. We determined that pro-inflammatory cytokines (TNFα, IFNγ, IL-17) upregulated CD13 mRNA in FLS, which may contribute to the increased CD13 in RA synovium and synovial fluid. Inhibition of CD13 function by either inhibitors of enzymatic activity or anti-CD13 antibodies resulted in decreased growth and diminished migration of FLS. This suggests that CD13 may be involved in the pathogenic hyperplasia of RA FLS. This data expands potential roles for CD13 in the pathogenesis of RA.

## Introduction

Aminopeptidase N/CD13 (EC 3.4.11.2), a metalloproteinase of the M1 family, is a Zn^+2^ dependent ectoenzyme that cleaves the N-terminal peptide from its substrates [1–4]. CD13 has been linked to the pathogenesis of a variety of immune-mediated conditions including rheumatoid arthritis (RA), scleroderma, psoriasis, and chronic graft-versus-host disease [2–8]. In addition to RA CD13 has also recently been implicated in osteoarthritis (OA) through a role on chondrocytes [9]. CD13 is primarily a cell surface molecule that was originally identified on myeloid cells [1], but is now known to be expressed by other cell types, including FLS [10]. It has also been identified in soluble fractions of biological fluids. CD13 is upregulated in RA synovial fluid compared to OA synovial fluid, normal human serum, or RA serum [10]. CD13 is also found in fibroblast like synoviocyte (FLS) culture supernatants, demonstrating that CD13 is released from FLS [10]. CD13 has been identified as a truncated soluble protein in human serum by Western blot; however, because CD13 is highly expressed on the cell surface, extracellular vesicles, which can reflect the protein composition of the cell surface, are another potential source of CD13 in cell free fractions [11,12].

Extracellular vesicles are composed of a variety of small vesicles including exosomes, microparticles, and apoptotic bodies. Apoptotic vesicles are released by dying cells and microparticles are released primarily from platelets, but exosomes can be released from a wide variety of cell types including FLS [13]. Exosomes are small (40–120 nm diameter) lipid bilayer vesicles that typically express a surface profile similar to that of the cells from which they are released [13]. CD13 has been previously demonstrated on exosomes from microglial cells and mast cells [14–17].

The goal of this study was to further understand the expression and function of CD13 on human RA FLS. We examined the effect of three pro-inflammatory cytokines linked to RA on CD13 expression by RA FLS, and determined how CD13 is released from FLS. We also examined the possibility that CD13 is present on exosomes or other extracellular vesicles derived from FLS and other human cell types, and measured soluble versus vesicle bound CD13 in sera, synovial fluids, and FLS culture supernatants. In addition we investigated possible autocrine effects of CD13 on RA FLS.

## Materials and Methods

### Cell Culture

All procedures involving specimens obtained from human subjects were performed under a protocol approved by the University of Michigan Institutional Review Board. FLS were cultured from human synovial tissue obtained at arthroplasty or synovectomy from RA joints by digestion with 1% collagenase and separation through a 70μM cell strainer [18]. FLS were uniformly positive for the FLS marker Cadherin-11. The diagnosis of RA required at least four of the seven 1987 American College of Rheumatology criteria [19]. FLS were maintained in Connaught Medical Research Laboratory (CMRL) medium (20% fetal bovine serum [FBS], 2mM L-glutamine, 1% penicillin/streptomycin) and were used between passages 4 and 10. To avoid the confounding effect of serum CD13, cultures were moved to serum free media Dulbecco's Modified Eagle's medium/F-12 with Peprogrow serum replacement (Peprotech, Rocky Hill, NJ) before harvesting. Some cultures were treated with protease inhibitors for 48 hours in serum free medium: pepstatin A (Sigma-Aldrich, St. Louis, MO), aprotinin (Sigma-Aldrich, St. Louis, MO), leupeptin (Sigma-Aldrich, St. Louis, MO), GM6001 InSolution (EMD Millipore, Darmstadt, Germany), or E-64 (Thermo Scientific, Waltham, MA). Other cultures were treated with cytokines: recombinant human interferon-γ (rhIFNγ, 1U/ml), recombinant human tumor necrosis factor-α (rhTNFα,10ng/ml), or recombinant human interleukin-17 (rhIL-17,10ng/ml) (Peprotech, Rocky Hill, NJ) for 0, 0.5, 1, 2, 6, 8, 12, 24, 48, or 72 hours in serum free medium.

### Sample Preparation

Synovial fluid samples were treated with 0.05% Hyaluronidase (bovine testis, Sigma-Aldrich, St. Louis, MO) one drop per 1 mL fluid for 5 min. Cells were lysed in cell lysis buffer (10% NP-40, 10% PMSF, 1% Iodoacetinimide, and 0.1% E-64 in TSA) for one hour on ice and spun to remove debris. FLS culture supernatants were concentrated by centrifugation through an Amicon Ultracel 30K filter (EMD Millipore, Darmstadt, Germany). Plasma was isolated from whole blood using heparin vacutainer tubes (BD biosciences, San Jose, CA).

### Exosome Isolation

Exosomes were isolated by serial ultracentrifugation [20]. Exosomes were isolated from either the supernatants of 3 flasks of confluent RA FLS, 10mls of plasma, or 1 ml of RA synovial fluid diluted 1:4 with PBS. Cells were pelleted out at 1500rpm for 5 minutes. Then the supernatants were cleared of heavier debris by centrifugation at 10,000xg for 30 minutes and 30,000xg for 1 hour. Exosomes were then obtained by ultra-centrifugation at 110,000xg for 4-20hours. Exosome pellets were washed in PBS at 110,000xg for 1.5 hours–overnight and resuspended in 1ml of PBS. Some exosomes were further purified using a density gradient, Optiprep (Sigma Aldrich, St. Louis, MO). Optiprep was diluted with PBS to produce the following layers: 5%, 10%, 15%, 20%, 30%, 40%, and 50% w/v (densities of 1.031, 1.050, 1.084, 1.110, 1.163, 1.215, and 1.268 g/ml). 500μl of extra cellular vesicle fractions were floated on the top of the density gradient and the gradients were centrifuged at 100,000xg for 1hour. Fractions were carefully pipetted off, washed with PBS, and centrifuged at 110,000xg for 2 hours. Pellets from the fractions were resuspended in 500μl PBS. Exosome size was measured by use of a NanoSight NS500 (Malvern Instruments, Salisbury, United Kingdom).

### CD13 Enzyme-Linked Immunosorbent Assay (ELISA)

High binding ELISA plates were coated with the anti-CD13 monoclonal antibody WM15 (Biolegend, San Diego, CA) in 0.1M carbonate buffer pH 9.5 overnight, and then blocked with 1x Animal Free Block (Vector Laboratories, Burlingame, CA) overnight. Samples were then applied to the plates either whole or diluted in block with 10mM EDTA. The standard curve was prepared using recombinant human CD13 (R&D Systems, Minneapolis, MN) in block with 10mM EDTA. 1D7 (591.1D7.34, University of Michigan Hybridoma Core), an anti-CD13 monoclonal antibody that was recently described [10], was biotinylated (Biotin-XX Microscale Protein Labeling Kit, Life Technologies, Carlsbad, CA) and applied overnight [21]. Streptavidin-HRP (Biolegend, San Diego, CA) was then added. Between steps plates were washed with PBS plus 0.05% Tween. The plates were visualized with TMB substrate (BD Biosciences, San Jose, CA), stopped with 2M H_2_SO_4_, and analyzed on a colorimetric plate reader.

### ELISA for the secretion of CD13 from RA FLS

To determine the role of tissue inhibitors of metalloproteinases (TIMPs) in the secretion of CD13, RA FLS (10×105 cells) were plated in 6-well plates in RPMI with 10% FBS. When RA FLS became 85% confluent, media was switched to RPMI containing 0.1% FBS. RA FLS were incubated with TIMP-1, TIMP-2 or both for 48 hours. ELISA was performed to determine the levels of soluble CD13 in the conditioned media. Conditioned media (100 μl/well) was added to 96-well plates (Thermo Scientific, USA) for 2 hours at room temperature. After blocking, anti-CD13 antibody, WM15, (10μg/ml, BioLegend) was added for 2 hours at 37°C. Anti-mouse IgG HRP-linked antibody (1:1000) was added for 1 hour. After adding the TMB substrate solution (BD Biosciences) and stop solution (2N H2SO4), the optical density (OD) was measured at 450 nm by a microplate reader (BIO-TEK, USA). We used FLS from three different RA patients. Both inhibitors were used at 0.6 μg/ml. These concentrations are recommended by the manufacture to inhibit MMPs.

### Aminopeptidase enzymatic activity

Aminopeptidase activity was measured by cleavage of L-Leucine-7-amido-4-methyl coumarin (L-leu-AMC, Sigma-Aldrich, St. Louis, MO) to release the fluorescent molecule AMC. A standard curve was constructed using AMC (Sigma-Aldrich, St. Louis, MO). The assay was run in 0.1 M Tris-HCl buffer (pH 8.0). Samples were incubated with the substrate at 37°C for one hour then read using a fluorescent plate reader at emission 450, excitation 365. Results were calculated as μM/hr of substrate cleaved.

### Western Blot

Exosome lysates, derived from exosomes isolated from RA FLS (15 μl), were boiled for 5 minutes in Laemmli’s sample buffer and, were subjected to 10%SDS- polyacrylamide gel electrophoresis (PAGE) followed by Western blot analysis. The proteins were electrophoretically transferred from the gel onto nitrocellulose membranes using a Tris-glycine buffer. To block nonspecific binding, membranes were incubated with 5% nonfat milk in Tris-buffered saline containing 0.01% Tween-20 (TBST) for 1 hour at room temperature. The blots were incubated in mouse antihuman Flotillin and CD9 (BD Biosciences) primary antibodies plus 5% nonfat milk in TBST at 4°C overnight. After washing with TBST, the blots were incubated with horseradish peroxidase–conjugated sheep anti–mouse with goat anti–rabbit IgG (1:3000) for 45 minutes at room temperature. An ECL detection system was used to identify specific protein bands.

### siRNA Knockdown

FLS were transfected by electroporation using an Amaxa Nucleofector and the nucleofector kit for dermal fibroblasts (NHDF, Lonza, Basel, Switzerland). In brief, FLS were released by trypsin and 5x10^5^ were transfected per condition. Cells were resuspended in transfection solution and either 300nM MMP14 short inhibitory RNA (siRNA)MMP1 siRNA ADAM15 siRNA, ADAM10 siRNA, ADAM17 siRNA (all, stealth RNAi [set of 3], Life Technologies, Carlsbad, CA), or 2μg pmaxGFP (Lonza, Basel, Switzerland) was added to each transfection cuvette. Cells were electroporated and transferred to flasks containing 20% CMRL. Transfected cells were grown for 5–7 days then transferred to serum free medium for 2 days before harvesting. Transfection of GFP control plasmid was measured by fluorescent microscopy (EVOSfl, AMG, Mill Creek, WA) and flow cytometry (BD Bisoceinces FACSCalibur, San Jose, CA). Knockdown efficiency was measured by qRT-PCR of MMP14 mRNA at the time of harvest for CD13 measurements.

### Confocal Microscopy

RA FLS were grown to 90% confluence on 8-well glass chamber slides. Cells were fixed with 1% Formalin and blocked with Fc block (10% human serum/10% mouse serum in PBS). Cells were incubated for 1hour at room temperature with anti-CD13-FITC (1D7) 1μg/100μl and anti-MMP14-PE (clone128527, R&D, Minneapolis, MN) 1.67μg/100μl or anti-CD90-PE 1μg/100μl (3E10, Biolegend, San Diego, CA). All experiments also included staining with MsIgG isotype controls (MsIg-FITC [eBiosciences, San Diego, CA], MsIg-PE [Biolegend, San Diego, CA]) at the same concentrations. The nuclei were counter stained with DAPI at 1μg/ml. Cells were mounted using Pro-gold anti-fade media (Life Technologies, Carlsbad, CA). Images were taken with an Olympus Fluo-View 500 confocal microscope system (University of Michigan Microscopy and Image Analysis Core) at 600x and1000x. All images were corrected for background, thresholds were determined by DAPI alone, MsIg-FITC alone, and MsIg-PE alone. Co-localization was analyzed with ImageJ using the plug-in “Colocalization”, by Pierre Bourdoncle, Institut Jacques Monod, Service Imagerie, Paris.

### Quantitative RT-PCR

mRNA was isolated from FLS (3 wells of a 6-well plate) using the RNAeasy Kit and Qiacube (Qiagen, Venlo, Netherlands). cDNA was prepared using a High Capacity cDNA Kit (Life Technologies, Carlsbad, CA). Quantitative reverse transcription polymerase chain reaction (qRT-PCR) was done using TaqMan Gene Expression Assays on a 7500 Real Time PCR System (Life Technologies, Carlsbad, CA).

### Flow cytometry

Fibroblasts were removed from flasks by 3mM EDTA in PBS. Cells were stained with MsIgG (negative control) or anti-CD13 (591.1D7.34) then goat anti-mouse IgG-Alexa fluor 488 (Life Technologies, Carlsbad, CA). Cytometry was performed on a BD Biosciences FACSCalibur. Gating was done to isolate the major cell population and exclude debris and dead cells.

### FLS Growth and Migration Assays

RA FLS were seeded on Essen Image Lock 96-well plates (Essen Bioscience, Ann Arbor, MI) overnight at either 3,000 cells/well for growth or 30,000 cells/well for migration. For growth the 20% FBS CMRL media was removed and cells washed 1x with PBS. 100μl of medium alone (control) or medium containing anti-CD3 (25 or 50ng/ml, OKT3, used as a non-reactive isotype control antibody in this experiment), anti-CD13 1D7 or WM15 (Biolegend, San Diego, CA) (25 or 50 ng/ml), or CD13 chemical inhibitors actinonin (Sigma-Aldrich, St. Louis, MO) or bestatin (Sigma-Aldrich, St. Louis, MO) (10μM and 50μM respectively) was added to the wells. Images and confluence data were collected using an Essen IncuCyte (Essen Bioscience, Ann Arbor, MI). For the scratch wound migration assay wounds were made via the Essen scratch wound tool in the seeded 96-well plate. Plates were then washed 2x with PBS and medium was added similar to the growth plates. Data were collected and the confluence or relative wound density calculated by the Essen IncuCyte.

### Statistics

Data are expressed as mean±standrad error of the mean (SEM). FLS data are expressed as a ratio of treated FLS to untreated FLS. Statistically significance was determined by unpaired student T-test.

## Results

### CD13 is found on Extracellular Vesicles Including Exosomes

**Fig 1:**
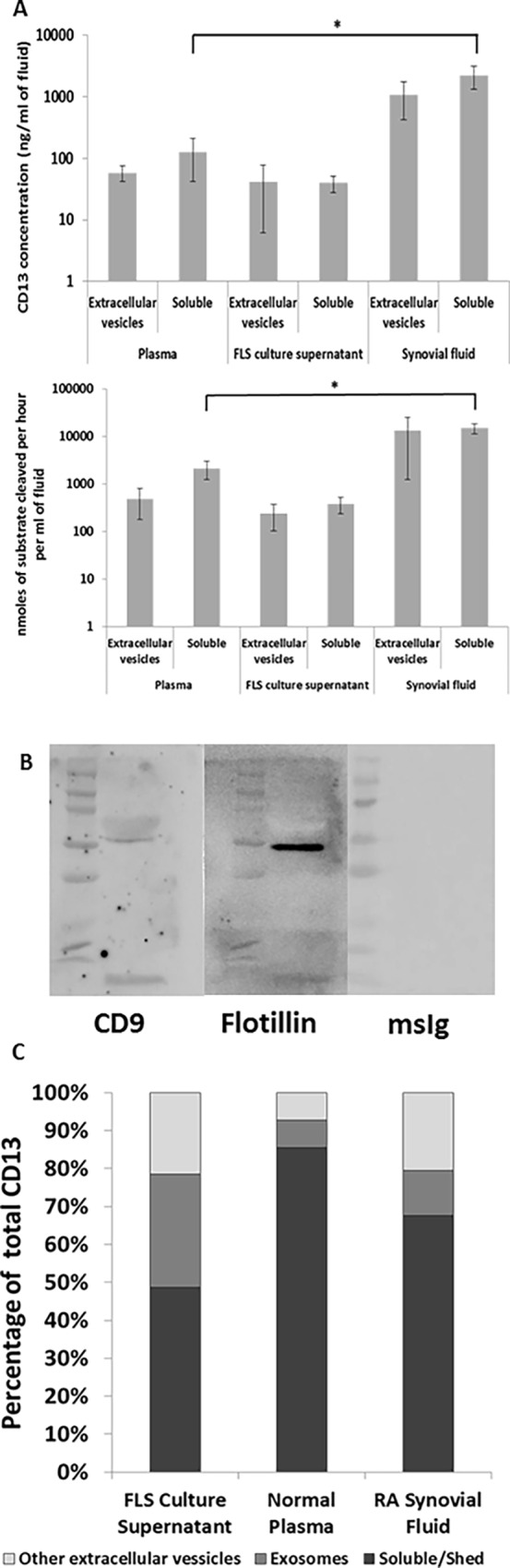
CD13 is found as a soluble protein and on extracellular vesicles. **(A)** Supernatants from 3 flasks of RA fibroblasts were concentrated through a 30K centrifugal filter. RA synovial fluid was diluted with PBS (4:1), and 10mls of plasma was obtained from a healthy individual. Vesicle fractions were isolated from the samples by serial centrifugation. Supernatants from the final centrifugation were collected as the soluble protein fraction. The vesicle pellet was resuspended in 1ml PBS. CD13 was measured by ELISA and aminopeptidase activity was analyzed through cleavage of L-leu-AMC. Data is normalized to concentration in original fluid. mean±SEM n≥3 **(B)** Exosomes were lysed and purity was confirmed by western blot for flotillin-1 and CD9. Single bands appeared at expected sizes with a weak band for CD9 and a strong band for flotillin-1 confirming exosomes from FLS. **(C)** A discontinuous optiprep gradient was created in seven fractions from 1.268g/ml to 1.031g/ml. 500ul of the resuspended vesicles was layered onto the top of the gradient. The loaded gradients were centrifuged at 100kg for one hour. Fractions were collected in reverse. Fractions were washed in PBS at 110kg for 2hr and the pellets were resuspended in 500ul PBS. Soluble CD13 is present in the first supernatant separation, exosomes are in fractions 3–5, and other extracellular vesicles are shown from the other fractions. CD13 was observed in all three fractions. Data was converted to percentage of total fluid CD13. % total n≥3

It has been previously shown that CD13 is present in synovial fluids, serum, and FLS culture supernatants [10]. However, the question remains as to whether CD13 in those fluids is a soluble molecule or bound on the surface of extracellular vesicles. To test the possibility that extracellular vesicles (EVs) also contain CD13, we isolated EVs and measured CD13 in the EV and soluble protein fractions. We used differential ultracentrifugation to isolate EVs corresponding to the density of exosomes. We identified CD13 in both soluble protein and EV fractions in plasma, RA FLS culture supernatant, and RA synovial fluid (Fig 1A). We used the specific CD13 sandwich ELISA assay to measure CD13 and the non-specific enzymatic assay to measure aminopeptidase activity. Plasma contained an average of 57.81±16.43ng/ml of CD13 on EVs with an activity of 487.41±308.51μM/hr, and 125.17±83.68ng/ml of soluble CD13 with an activity of 2134.83±884.81μM/hr. FLS culture supernatant contained an average of 41.89±35.76 ng/ml of CD13 on EVs with an activity of 241.00±137.01μM/hr, and 39.71±11.91ng/ml of soluble CD13 with an activity of 376.13±143.52μM/hr. RA synovial fluid contained an average of 1074.68±652.41ng/ml of CD13 that was associated with EVs with an activity of 13309.33±12061.47μM/hr, and 2236.71±902.68ng/ml of soluble CD13 with an activity of 14960.44±3739.09μM/hr.A significant difference was observed between the levels of CD13 (p = 0.039) and aminopeptidase activity (p = 0.012) in the soluble fractions of plasma compared to RA synovial fluid.

Although differential centrifugation is a suitable protocol for isolation of exosomes, there may be other contaminants of similar density (including apoptotic bodies and protein aggregates). We analyzed the extracellular vesicles by NanoSight. Exosomes were defined as being of size 30-130nm which is consistent with the expected size of exosomes identified by the NanoSight [22,23]. Isolation of exosomes from FLS was confirmed by western blot for flotillin-1 and CD9. Consistent with exosomes originating from FLS we saw a strong single band for flotillin-1 and a weak band for CD9 [24]. Exosomes defined by size made up 71.8% of the EVs in RA FLS culture supernatant (mode 85.5 nm), 85.7% of the EV’s in normal human plasma (mode 58.7 nm), and 57% of the EV’s in RA synovial fluid (mode 110.1 nm). An example of the NanoSight data is provided in S1A Fig. To further define which extracellular structures contain CD13, we divided the EV fraction over a discontinuous Optiprep density gradient with seven fractions from 1.268g/ml to 1.031 g/ml, to divide the EV fractions more specifically by density. We visually confirmed a band at the density gradient between fractions 4 and 5 where exosomes would be expected (density between 1.084g/ml and 1.163 g/ml fractions 3–5). We also found CD13 present by ELISA in all seven gradient fractions as well as soluble protein fractions (S1B Fig). The three types of fluids that were analyzed (RA FLS culture supernatant, healthy control plasma, and RA synovial fluid) each exhibited a distinct pattern of CD13 localization (Fig 1C). FLS culture supernatant was the most balanced with an average of 48.67% soluble CD13, 29.76% exosomal CD13, and 21.58% other EV CD13. Healthy control plasma was predominantly soluble with 85.60% soluble CD13, 7.09% exosomal, and 7.31% CD13 on other EVs. The RA synovial fluid contained 67.55% soluble, 11.92% exosomal, and 20.53% from other EVs.

### Metalloproteinases cleave CD13 from FLS

**Fig 2:**
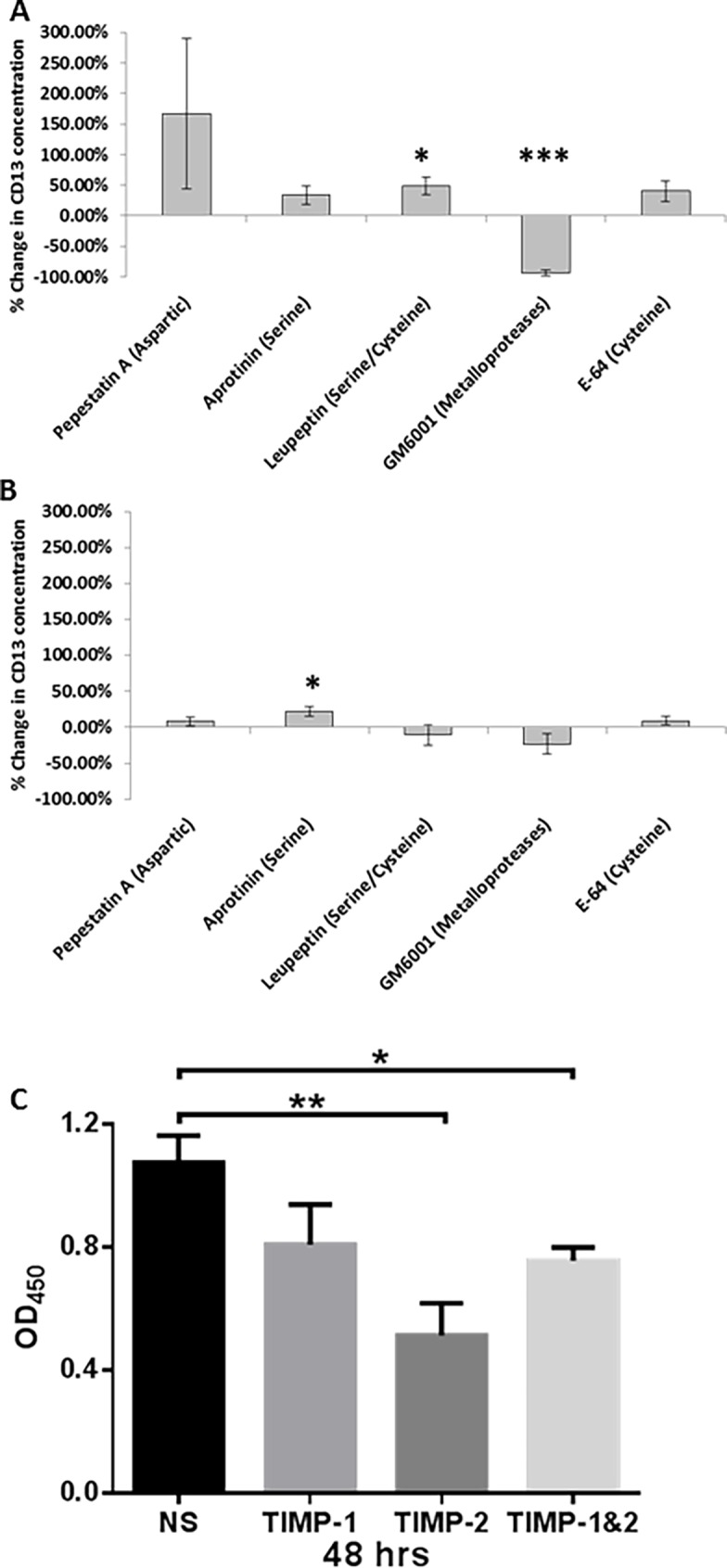
Metalloproteinases cleave CD13 from the surface of FLS. Five different protease inhibitors were added to FLS cultures covering all classes of proteases. **(A)** The only inhibitor to decrease shedding of CD13 into the supernatant of the cultures was GM6001. **(B)** No significant decreases were seen in cell lysate CD13 concentrations. Cultures were incubated with serum free media containing protease inhibitors for 48 hours: Pepstatin A (aspartic) 10μM, Aprotinin (serine) 100nM, Leupeptin (serine/cysteine) 10μM, GM6001 (metalloproteinase) 25μM, and E-64 (cysteine) 10μM. **(C)** TIMP-2 (0.6μg/ml) inhibited the secretion of CD13 from RA FLS while TIMP-1(0.6μg/ml) did not. We used FLS from 3 different RA patients. Secretion was measured by ELISA and optical density (OD) of the samples was measured. (n = 3) mean of % change ± SEM *p≤0.05 ***p≤0.0001

Since CD13 exists as a soluble molecule in cell-free portions of biological fluids separate from vesicle-associated CD13, soluble CD13 must be released from cells. Since soluble CD13 was found in FLS culture supernatants, we explored how CD13 was released from FLS. CD13 is highly expressed on the cell surface of FLS and therefore could be shed. To test this mechanism we added various protease inhibitors to FLS cultures, including: pepstatin A (aspartic), aprotinin (serine), leupeptin (serine/cysteine), GM6001 (metalloproteinase), and E-64 (cysteine), and found that only one, GM6001, blocked CD13 release from FLS. All inhibitors were used at established working concentrations [25,26]. All of these inhibitors are known to have low toxicity, and we did not observe any significant cell death as measured by trypan blue staining upon culture harvest. Therefore pharmacologic toxicity is not contributing to the results [27,28]. In all cases total CD13 concentration was higher in the cell lysate than in the cell culture supernatant. GM6001significantly reduced CD13 protein found in the supernatant by 93.62±4.78%, p≤0.0001 (Fig 2A). Leupeptin led to a significant (p≤0.05) increase (48.40±14.29%) in CD13 released. To confirm that the inhibitors were affecting cleavage and not expression, CD13 was also measured in the FLS cell lysates. No significant difference was observed with GM6001; however, aprotinin induced a significant increase (22.17±6.18%, p≤0.05) in CD13 expression (Fig 2B). These results indicate that CD13 is cleaved from FLS by metalloproteinases.

Tissue inhibitors of metalloproteinases (TIMPs) were used to determine whether CD13 was cleaved by soluble versus membrane bound matrix metalloproteinases. We found a significant decrease in CD13 secretion (measured by direct CD13 ELISA) in the conditioned media collected from RA FLS incubated with TIMP-2 for 48 hours (p<0.05). We did not find this decrease when RA FLS were incubated with TIMP-1, suggesting that metalloproteinases inhibited byTIMP-2 contribute to the secretion of CD13 from FLS (Fig 2C). The results were confirmed by sandwich ELISA (data not shown). This supports a membrane-type matrix metalloproteinase as being involved in the cleavage of CD13.

### MMP14/MT1-MMP cleaves CD13 from FLS

**Fig 3:**
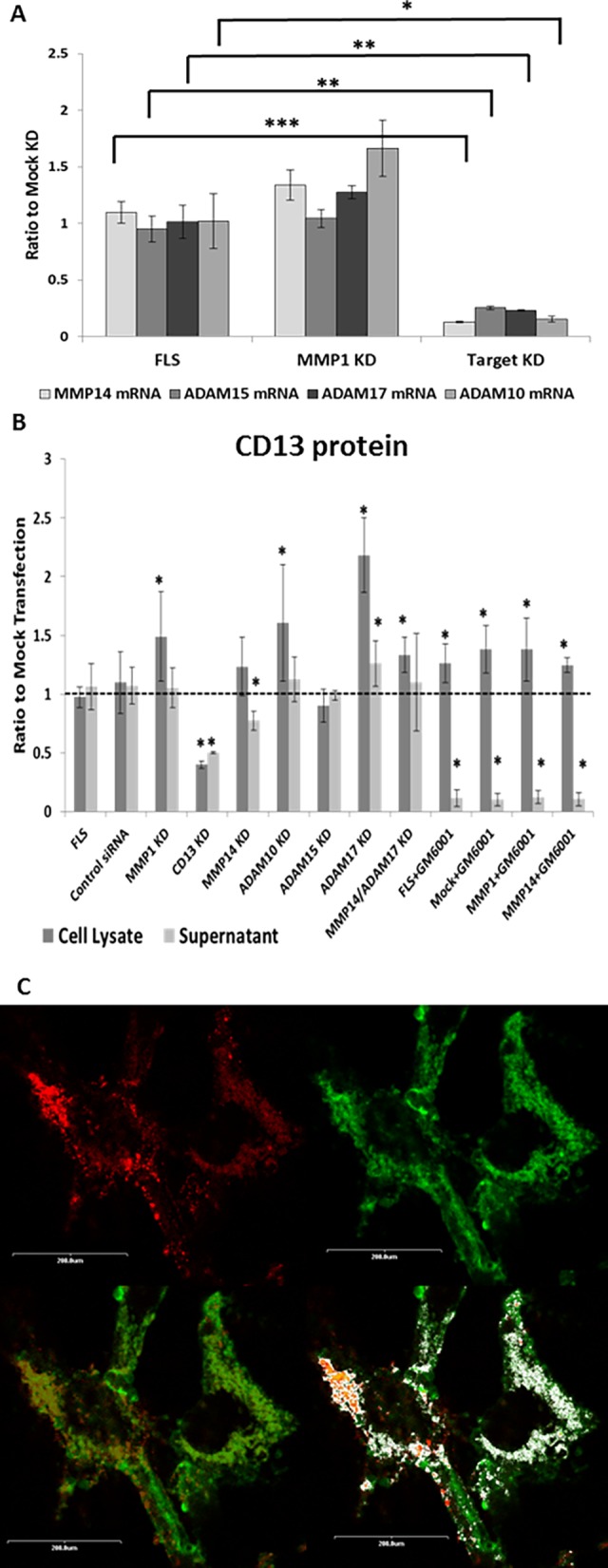
MMP14 knockdown partially inhibits the shedding of CD13 from FLS. **(A)** MMP14 mRNA was almost completely removed by transfection with MMP14 siRNA. ADAM10, ADAM15, and ADAM17 knockdown constructs each also almost completely removed their respective mRNAs with appropriate siRNA transfection. None of the siRNAs (MMP14, ADAM10, ADAM15, or ADAM17) had an off target effect on MMP1. n = 3 (**B)** Shedding of CD13 into FLS culture supernatant was inhibited by knockdown of MMP14. Further inhibition was seen with GM6001. CD13 in FLS lysate was not changed by knockdown of MMP14, and significantly increased in GM6001 treated FLS. FLS were grown to confluence then transfected with siRNA using an Amaxa nucleofector kit. Cells were grown to 75% confluence then switched to serum free growth media (Peprogrow) supplemented with 1ng/ml IL-1 and 10ng/ml TNFα for 48 hours with or without 25μM GM6001. **(C)** Cells were incubated for 1hour at room temperature with anti-CD13-FITC (1D7) 1μg/100μl and anti-MMP14-PE (128527) at 1.67μg/100μl. Figures shown were taken at 1000x. The four panels show: top left, MMP14 alone (red); top right, CD13 alone (green); bottom left, co-localization (co-localization in yellow); and bottom right, co-localization analysis by ImageJ (white = overlapping red and green pixels). All images were background corrected using DAPI alone and MsIg-FITC and MsIg-PE for threshold limits. Representative of n = 6 *p≤0.05 **p≤0.005 ***p≤0.0001 mean±SEM (n≥2)

Matrix metalloproteinase 14 (MMP14/MT1-MMP) is a membrane-type metalloproteinase on FLS that is critical to FLS invasion of collagenous structures [29,30]. MMP14 is up-regulated on RA FLS, and we hypothesized that the metalloproteinase that cleaves CD13 from FLS is MMP14 [30–32]. We therefore knocked down MMP14 in RA FLS using siRNA. Fig 3A shows an example of successful knockdown of MMP14. Fold change for MMP14 over GAPDH and ratio to mock control (ΔΔCt) of FLS was 1.10±0.10, and was decreased to 0.13±0.0064 with addition of MMP14 siRNA. ADAM15 siRNA, ADAM10 siRNA, ADAM17 siRNA also significantly knocked down the expression of their respective mRNAs (Fig 3A). Moreover no off target effects were seen on MMP1 mRNA. GFP plasmid transfection was used to determine transfection efficiency. Higher fluorescence was observed with both flow cytometry and fluorescent microscopy over mock transfection controls. Green fluorescence measured by flow cytometry increased from 6.77 mean fluorescent intensity (MFI) in the negative control (mock transfected) to 111.23 MFI in the transfected FLS.

Knockdown (KD) of MMP14 significantly decreased the CD13 released from FLS (Fig 3B). Samples were normalized to mock transfection in order to compare between experiments (n≥3) and CD13 in mock transfection is shown on the graph as a line at one. The only significant difference in the siRNA KD of test groups (MMP14, ADAM15, ADAM17, ADAM10) was seen with MMP14 (n = 14) in which case the KD cells released CD13 at 0.78±0.08 of control levels, p = 0.0031 (Fig 3B). CD13KD was used as a positive control, and MMP1 KD was used as a negative control for membrane anchored metalloproteinases. To confirm we were measuring cleavage and not a decrease in CD13 expression, CD13 protein was also measured in cell lysates. Knockdown of MMP14 did not significantly alter cellular CD13; MMP14 KD ratio over mock was 1.24±0.25 (Fig 3B). Because MMP14 KD resulted in an average decrease of only 23% in supernatant CD13, this indicates that more than one protease can cleave CD13. To confirm that the other CD13-releasing protease(s) are also metalloproteinases, GM6001 was added to MMP14 KD cultures (Fig 3B, n = 2). Similar to previous results, GM6001 prevented the release of CD13 into the culture supernatant (p = 1.42x10^-9^). Addition of GM6001 to MMP14 KD cultures also decreased supernatant CD13 to a level significantly lower than MMP14 KD without GM6001 (p = 3.21x10^-9^). In the lysates of GM6001 treated cells CD13 was significantly increased (p = 0.0070). Single knockdowns of several other possible candidates (ADAM15, ADAM10, or ADAM17) did not decrease CD13 release from FLS and ADAM17 KD did not add to the effect of MMP14 KD. ADAM17 KD actually led to a significant increase in CD13 expression in both the cell lysate and culture supernatant (p = 2.01x10^-8^ and p = 0.028 respectively). It should be noted that siRNA KD of CD13 itself (n = 2) resulted in an approximate 50% decrease relative to mock transfected: cell lysate 0.40±0.031, p = 1.18x10^-17^; culture supernatant 0.50±0.0083, p = 7.58x10^-26^. This shows that additional metalloproteinases in addition to MMP14 are involved in the cleavage of CD13, but does not identify any one enzyme of equivalent importance to MMP14 in the shedding of CD13.

To confirm the role of MMP14 in the release of CD13, confocal microscopy was used to look for co-localization on the surface of RA FLS. Cells were stained with DAPI for nuclei (blue, not shown), anti-CD13-FITC (green), and anti-MMP14-PE (red). We observed predominant co-localization of CD13 and MMP14 on all tested FLS, with lesser areas of individual staining. Images shown in Fig 3C are representative of n = 6. The bottom left hand picture shows CD13 (green) and MMP14 (red) signals overlapping on FLS to form yellow. The bottom right hand picture shows a computer analysis, with co-localized pixels in white and individual color areas in green or red. Staining of a second FLS line is shown in S2 Fig with a CD90 control. The results indicate that MMP14 co-localizes with CD13 on FLS and contributes to cleaving of CD13 from the FLS membrane to generate its soluble form. However, other undetermined metalloproteinases (likely redundantly and in combination) also contribute to CD13 cleavage.

### Regulation of CD13 expression on FLS

**Fig 4:**
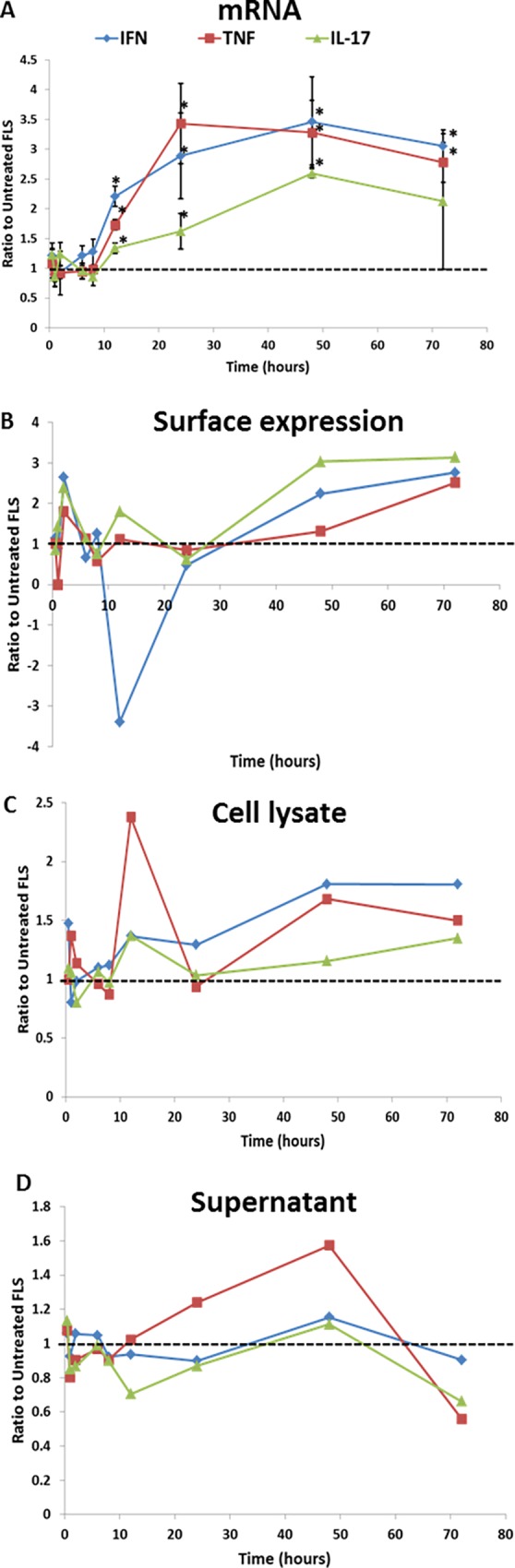
CD13 is upregulated in FLS at the mRNA level but the effects on CD13 protein levels are varied. FLS were stimulated over a time course of 0–72 hours with IFNγ (1000U/ml), TNFα (10ng/ml), or IL-17 (10ng/ml). Cells were harvested and processed for either mRNA (A), surface expression (B), total cell lysate CD13 (C), or total CD13 in supernatant (D). mRNA was measured by qRT-PCR. CD13 was measured on the surface with anti-CD13 (1D7) and flow cytometry. Cell lysate and supernatant CD13 concentrations were measured by CD13 ELISA. Gating was done to isolate the major cell population and exclude debris and dead cells. Data is expressed as a ratio to unstimulated FLS at the same time point in either ΔΔCt normalized to GAPD (mRNA), mean fluorescent intensity corrected for background florescence with MsIg staiming (surface), or CD13 concentration in lysate or supernatant. A n = 3 B-D n = 1 *p≤0.05

CD13 is present at much higher levels in RA synovial fluid compared to OA. However, cultured RA and OA FLS expressed similar amounts of CD13 [10]. One possible explanation for this observation is that the pro-inflammatory cytokines in the RA joint could contribute to upregulation of CD13 production by FLS. Cultured RA FLS were treated with IFNγ, TNFα, or IL-17 over a time course from 0 to 72 hours. CD13 mRNA, as measured by qRT-PCR, was upregulated by all three cytokines with a peak expression around 48 hours (Fig 4A). Data shown are a ratio of cytokine treated FLS CD13 mRNA to untreated FLS CD13 mRNA at the same time point. IFNγ and TNFα-exposed cells were significantly upregulated (p≤0.05) at 12, 24, 48 and 72 hours, and the IL-17effect was significant at 8, 12, 24, and 48 hours. However, expression of CD13 protein exhibited variability and fluctuations that did not match the change in CD13 mRNA. Fig 4B shows the results of one cell line examined by flow cytometry with staining by anti-CD13 (1D7). Fig 4C shows CD13 (ng/ml) in total cell lysate, and Fig 4D shows CD13 (ng/ml) in FLS cell supernatant (both measured by ELISA). Other FLS lines showed comparable fluctuations, which may in part be explained by shifting of CD13 between various cellular and extracellular compartments.

### CD13 aids in growth and migration of RA FLS

**Fig 5:**
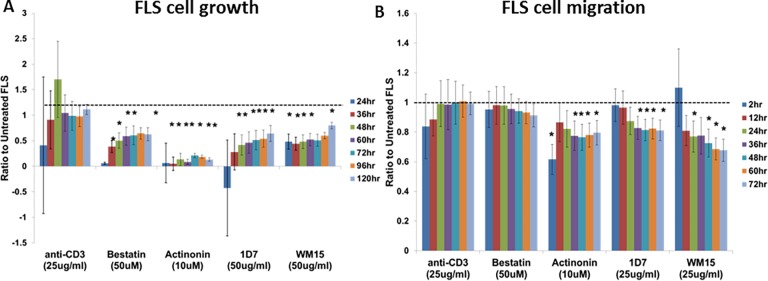
CD13 chemical inhibitors or antibodies significantly slow growth and migration of RA FLS *in vitro*. FLS were seeded on 96-well plates overnight. An Essen Incucyte system was used for both growth (**A**) and migration (**B**) assays. Growth was calculated as the difference in percent confluence from time 0. Migration was measured in a scratch wound assay using relative wound density. All data is expressed as a ratio to untreated FLS of the same cell line. mean±SEM n≥4 (*p≤0.05)

One mechanism that could account for fluctuating levels of soluble CD13 in FLS culture media would be uptake of CD13 by the RA FLS in an autocrine manner. To determine possible functions for CD13 on FLS we examined the effect of anti-CD13 antibodies (WM15 or 1D7) or CD13 chemical inhibitors (Bestatin or Actinonin) on RA FLS growth and migration. Anti-CD3 was used as a negative, isotype control. We observed a significant slowing of cell growth with both CD13 inhibitors and both anti-CD13 antibodies (Fig 5A). Data are expressed as the change from 0 hr as a ratio to untreated FLS of the same cell line at the same time point. The significant (p≤0.05) slowing of growth was observed primarily between 24 hours and 120 hours with actinonin being the strongest inhibitor of cell proliferation. A significant decrease (p≤0.05) was also seen in RA FLS migration in a wound healing assay in the presence of actinonin, WM15 or 1D7 primarily from 36 hours to 72 hours (Fig 5B). These data demonstrate an autocrine effect of CD13 on FLS. Examples of scratch wound images for anti-CD3 control, actinonin, and 1D7 are shown in S3 Fig.

## Discussion

Recent evidence indicates an important role for CD13 in RA as a T cell chemoattractant [10], and the data presented in this report suggests additional roles for CD13 in the pathogenesis of RA. CD13 has been found in the cell free portions of various biological fluids, including FLS culture supernatant and synovial fluid [6,10,33,34]. There are three possible mechanisms by which FLS may release CD13: secretion through exocytosis of sCD13, protease-mediated cleavage from the cell surface, and secretion of CD13 on the surface of extracellular vesicles such as exosomes. We performed differential ultracentrifugation to differentiate between vesicle associated and soluble CD13, and we found that CD13 was present both on exosomes and as a soluble molecule. We hypothesized that as a strongly expressed cell surface structure, cleavage of CD13 was more likely than secretion of sCD13. This notion is supported by the observation that sCD13 identified in serum is truncated and lacks the intracellular and transmembrane domains, suggesting cleavage from the cell membrane [12]. We examined this mechanism using inhibitors specific for different classes of proteases: pepstatin A (aspartic), aprotinin (serine), leupeptin (serine/cysteine), GM6001 (metalloproteinases), and E64 (cysteine). Our data indicate that CD13 is cleaved from FLS by metalloproteinases (Fig 2A). While there are several sub-classes, there are two main groups of metalloproteinases, matrix metalloproteinases (MMPs) and a disintegrin and metalloproteinase (ADAMs). Transmembrane proteases, which are known to participate in cleavage and release of proteins anchored in the membrane, are most likely responsible for release of CD13. Several of these proteases are members of the metalloproteinase family (MMPs14,15,16,17 and many ADAMs). Furthermore our experiments with TIMPs indicate that membrane-type matrix metalloprotienases (MT-MMPs) are a more likely to be mediating shedding of CD13 than soluble MMPs. Thus TIMP-1, which poorly inhibits MT-MMPs but inhibits soluble MMPs effectively, did not significantly suppress cleavage of CD13, while TIMP-2, which inhibits both MT-MMPs and soluble MMPs, did significantly decrease cleavage of CD13 (Fig 2C) [35].

Of the membrane-type MMPs, MMP14 is found at the highest amount on the surface of RA FLS [36,37]. In RA, MMP14 has already been linked to matrix degradation by FLS and osteoclast-mediated bone resorption [37]. Multiple studies have shown that of the MMPs expressed by synoviocytes, MMP14 in particular is important as a type I and type II collagenase and is essential for invasion of cartilage by FLS[30–32,38]. Moreover, we found that siRNA inhibition of MMP14 resulted in a significant decrease in CD13 cleaved from FLS (Fig 3C). While MMP14 KD results in an approximate 23% decrease in CD13 in the supernatant, MMP14 is likely primarily involved in the release of soluble CD13. Soluble CD13 accounted for around half of the CD13 in FLS culture supernatants with the other half from EVs (Fig 1C). Thus, knockdown of MMP14 might be expected to only partially affect release of CD13 from FLS, if MMP14 controlled cleavage of CD13 from the FLS membrane, but not release in EVs. In addition, inhibition of all metalloproteinases by GM001 further reduced the CD13 released into the supernatant. The data are consistent with roles for various MMPs in release of CD13 on EVs, and a primary role for MMP14 in cleavage of membrane CD13 from the cell surface.

Many members of the metalloproteinase family (and especially ADAMs) have the same or similar substrates, and multiple metalloproteinases can be involved in the same biological functions [39–44]. Even though collagenolytic activity is the best characterized example of a shared substrate, with most MMPs demonstrating this function, the similarity of cleavage sites and activity may carry over to other substrates [29]. It is possible that other membrane bound MMPs (MMP15, 16, or 17) are also involved in CD13 shedding. MMP15/MT2-MMP mRNA has previously been found in RA synoviocytes, and MMP16/MT3-MMP has been found on synovial tissue biopsies [30,37]. However, we found very little mRNA of either MMP15 or MMP16 in our RA FLS lines (data not shown). The other possible group of CD13 sheddases is the ADAMs. This is supported by the observation that some soluble CD13 remains even after inhibition with TIMP-2 (Fig 2C), given that TIMP-3 is the primary suppressor of ADAMs [35]. The fact that TIMP-1 and TIMP-2 combined did not completely inhibit shedding of CD13 would indicate that metalloproteinases other than MMPs are involved, specifically ADAM family members. In particular ADAM17, ADAM15, and ADAM10 have been linked to shedding of various proteins [40,44,45]. ADAM17 has also been suggested to interact with CD13 on the surface of myeloid leukemia cells [21]. ADAM15 has been found to be up-regulated in RA synovium compared to OA, is constitutively expressed by RA FLS, and has been linked to angiogenesis and FLS migration[46–48]. Single knockdown of ADAMs 10, 15, or 17 did not result in a decrease of CD13 in the culture supernatant (Fig 3B). Based on our observations we conclude that metalloproteinases cleave CD13 from the FLS membrane, with MMP14 being the primary sheddase while multiple other metalloproteinases likely act together to also shed CD13. In addition, metalloproteinases may act in an as yet poorly-characterized role in the release of CD13^+^ EVs from FLS. This notion is supported by a report that GM6001 inhibited the release of exosomes from endothelial cells [49].

To confirm MMP14 as a cleaver of CD13, we also looked for co-localization of CD13 and MMP14 on FLS. CD13 and MMP14 have previously been found in similar cell surface domains, but their proximity has not been determined. Both CD13 (FLS) and MMP14 (breast carcinoma and glioma cells) have been found in caveolae-enriched lipid rafts [50,51]. We show that, on the surface of FLS, CD13 and MMP do co-localize and in some cells we have observed a punctate pattern, which may be indicative of inclusion into lipid raft structures (Fig 3C, S1 Fig.). Overall these data indicate that CD13 and MMP14 localize in similar areas on the FLS cell surface, and therefore support the hypothesis that MMP14 may cleave CD13.

We identified CD13 in vesicle fractions in plasma, synovial fluid, and FLS culture supernatant and as a soluble molecule (Fig 1A). Nanosight counting revealed a predominance of exosome sized vesicles in the CD13^+^ vesicle fractions. Differential ultracentrifugation revealed that CD13 was associated with vesicles at a density similar to that of exosomes. Further density separation identified CD13 at densities from 1.268g/ml to 1.031 g/ml, indicating its presence on exosomes as well as other extracellular vesicles of similar density (Fig 1B). One problem with the differential centrifugation method is that it can isolate other EVs or large protein aggregates of similar density to exosomes. However, the additional separation by density gradient can distinguish between exosomes, other EVs, and protein aggregates. Apoptotic blebs float at about a >1.23g/ml density while exosomes float at 1.10–1.21g/ml [52]. Our results demonstrate that CD13 is present as both a soluble molecule and on extracellular vesicles derived from FLS.

CD13 represents a significant portion of the T cell chemotactic ability of RA synovial fluid [10]. Once in the joint, T cells are known to activate RA FLS through cell-cell interactions and the release of pro-inflammatory cytokines [53–55]. This activation can result in greater production of chemokines by the FLS resulting in a self-perpetuating, pro-inflammatory cycle [55]. We previously observed that while there are no differences in CD13 expression between OA and RA FLS in culture there is significantly more CD13 in RA than in OA synovial fluid [10]. One hypothesis is that pro-inflammatory cytokines produced by invading cells (T cells/monocytes) up-regulate CD13 in the RA synovium, but that under culture conditions this up-regulation reverts to a baseline level. To determine whether CD13 expression could be a part of this inflammatory loop, we examined the effect of three pro-inflammatory cytokines on CD13 expression by FLS. CD13 mRNA was upregulated by IFNγ, TNFα, and IL-17 in FLS. However, the intensity of CD13 protein expression on the FLS cell surface did not match this regulation pattern. Even before the mRNA was upregulated (peak around 48 hours) the cytokines induced fluctuations in cell surface, total cell lysate, and supernatant CD13 (Fig 4). Overall, we can conclude that IFNγ, TNFα, and IL-17 up-regulate CD13 mRNA, and also change both protein expression and localization, with distinct kinetics in individual RA FLS lines. A high degree of variability was seen within and between cell lines indicating that this is a complex and dynamic process. Variation in intracellular localization and internalization of CD13 has been suggested in other publications. CD13 can mediate phagocytosis in monocytic lineage cells thereby also undergoing internalization [56,57]. In mice it has been shown that CD13 negatively regulates inflammation through co-internalization of TLR4 and CD13, thereby negatively regulating TLR4 signaling [58]. While this may or may not be relevant in human FLS, it suggests that CD13 localization (cell surface versus other) can be important in control of inflammation. Thus CD13 may be crucial to the fine balance of inflammation through both positive and negative regulation of inflammatory signals depending on its localization. For example, on the cell surface it may act to down regulate TLR4 signaling while as a soluble molecule it acts as a T cell chemoattractant [10,58]. CD13 has been shown to be present in caveolae lipid rafts in both FLS and monocytes which may suggest a mechanism for CD13 internalization that may contribute to inflammatory regulation in addition to the shedding that we have demonstrated [51,59].

Another component of disease pathology in RA is aggressive outgrowth and migration of FLS, manifested clinically as synovial hyperplasia. Previous data has implicated CD13 in the migration but not proliferation of dermal fibroblasts [60]. Our data indicate a role for CD13 in the growth and migration of RA FLS (Fig 5). However, it is uncertain whether this is dependent on soluble CD13 or cell surface CD13. We found that while inhibitors of CD13 enzymatic activity and anti-CD13 antibodies each inhibited FLS proliferation and migration, additional rhCD13 did not consistently affect the RA FLS (data not shown). This may reflect the large amount of CD13 produced by the FLS that can act in an autocrine fashion. Because chemical inhibitors of CD13 enzymatic activity, bestatin and actinonin, inhibit FLS proliferation and actinonin inhibits migration it appears that CD13 enzymatic activity may be necessary for these functions. Also, the specific antibody WM15, which inhibits CD13’s enzymatic activity, inhibited proliferation and migration. However, an anti-CD13 antibody that does not inhibit enzymatic activity, 1D7, also inhibited FLS growth and migration [10]. The most likely explanation is steric hindrance. 1D7 does not block cleavage of the small molecule L-leu-AMC in the CD13 aminopeptidase activity assay; however, it may block the ability of CD13 to associate with larger substrates on FLS, thereby indirectly but effectively blocking the enzymatic activity and cell growth or migration. The role of CD13’s enzymatic activity in cell motility is also supported by data using endothelial cells [61].

Bestatin was not found to inhibit FLS migration though it did inhibit FLS growth. This may be due to the fact that bestatin is not specific for CD13. Bestatin may exert effects on other peptidases that counteract the effect on CD13. However, the functional role of CD13 is confirmed by the specific anti-CD13 antibodies. These results demonstrate another potential pathogenic role for CD13 in RA through its effects on RA FLS in addition to its previously published function as a T cell chemoattractant in RA.

Overall, current and prior results point to roles for CD13 in the pro-inflammatory milieu of the RA synovium. CD13 is upregulated by pro-inflammatory cytokines, and is released from FLS. A recent report demonstrated that CD13 is a chemotactic molecule for T cells phenotypically similar to RA synovial T cells [10]. Targeting of the molecules responsible for the release of CD13 (such as MMP14) may be a point of regulation for inflammatory diseases such as RA.

## Supporting Information

S1 Fig **(A)** An example of NanoSight analysis. The left panel shows a single run of synovial fluid extracellular vesicles with concentration on the y-axis and size of particle on the x-axis. The background shows a frame of the count. Red crosses are a counted particle. The right panel shows an average of all runs for a synovial fluid with concentration on the y-axis and size of particle on the x-axis. The dashed lines show approximate exosome size. **(B)** A discontinuous Optiprep gradient was created in seven fractions from 1.268g/ml to 1.031g/ml. 500ul of the resuspended vesicles was layered onto the top of the gradient. The loaded gradients were centrifuged at 100kg for one hour. Fractions were collected in reverse. Fractions were washed in PBS at 110kg for 2hr and the pellets were resuspended in 500ul PBS.
(TIF)

S2 Fig Fluorescent staining of FLS shows co-localization of CD13 and MMP14. RA FLS were grown to 90% confluence on 8-well glass chamber slides. Cells were fixed with 1% Formalin and blocked with Fc block (10% human serum/10% mouse serum in PBS). Cells were incubated for 1hour at room temperature with **(A)** anti-CD13-FITC (1D7) 1μg/100μl or **(G)** anti-CD90-FITC 1μg/100μl and **(B and H)** anti-MMP14-PE (128527) at 1.67μg/100μl (appropriate isotype controls and single staining were also done, not shown). The nuclei were counter stained with **(C and I)** DAPI at 1μg/ml. Overlapping signals are shown in **D** and **J**. Cells were mounted using anti-fade media. Confocal microscopy was performed using an Olympus microscope. All images corrected for background–thresholds determined by DAPI alone, MsIg-FITC alone, and MsIg-PE alone. Co-localization analysis was run using an ImageJ add-in, red and green pixels that co-localize are shown in white **(E, CD13-MMP14; K, CD13-CD90)** and the scatter plots of co-localization are shown in **F** and **L** respectively. Representative of n = 6
(TIF)

S3 Fig Examples of scratch wound images show decrease in FLS migration with actinonin and anti-CD13 (1D7) compared to irrelevant isotype control (anti-CD3). FLS were seeded on 96-well plates overnight. An Essen Incucyte system was used for scratch wounds and migration measurements. Migration was measured in a scratch wound assay using relative wound density. Representative of n≥4.
(TIF)
