# Supplementary figures and images for: Localization, Shedding, Regulation and Function of Aminopeptidase N/CD13 on Fibroblast like Synoviocytes
Source: PLoS One. 2016 Sep 22;11(9):e0162008. doi: 10.1371/journal.pone.0162008 (PMC5033571; doi:10.1371/journal.pone.0162008)

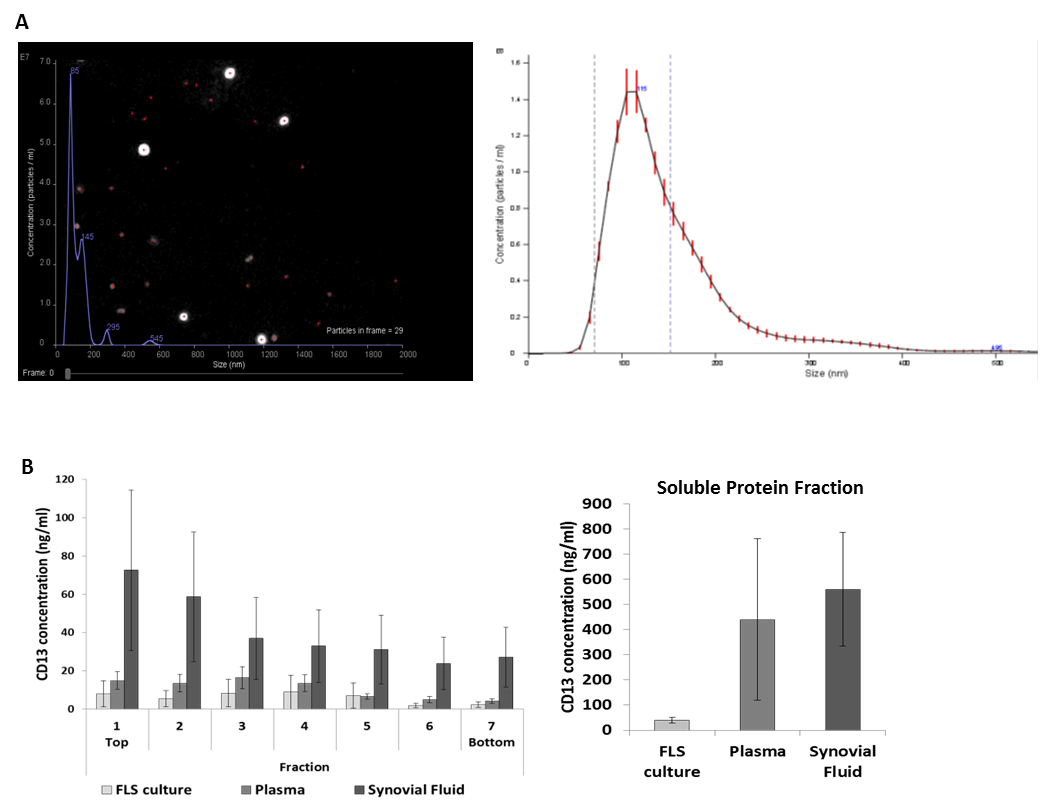

Supplement: S1 Fig — (A) An example of NanoSight analysis. The left panel shows a single run of synovial fluid extracellular vesicles with concentration on the y-axis and size of particle on the x-axis. The background shows a frame of the count. Red crosses are a counted particle. The right panel shows an average of all runs for a synovial fluid with concentration on the y-axis and size of particle on the x-axis. The dashed lines show approximate exosome size. (B) A discontinuous Optiprep gradient was created in seven fractions from 1.268g/ml to 1.031g/ml. 500ul of the resuspended vesicles was layered onto the top of the gradient. The loaded gradients were centrifuged at 100kg for one hour. Fractions were collected in reverse. Fractions were washed in PBS at 110kg for 2hr and the pellets were resuspended in 500ul PBS. (TIF) [file pone.0162008.s001.tif]

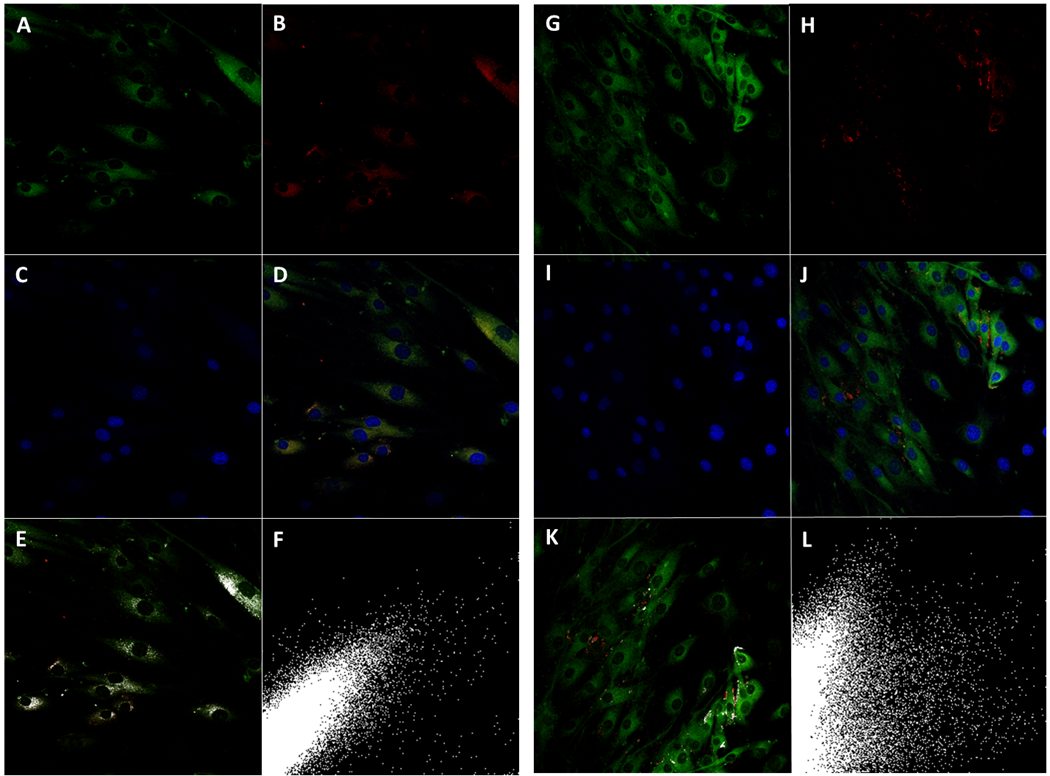

Supplement: S2 Fig — RA FLS were grown to 90% confluence on 8-well glass chamber slides. Cells were fixed with 1% Formalin and blocked with Fc block (10% human serum/10% mouse serum in PBS). Cells were incubated for 1hour at room temperature with (A) anti-CD13-FITC (1D7) 1μg/100μl or (G) anti-CD90-FITC 1μg/100μl and (B and H) anti-MMP14-PE (128527) at 1.67μg/100μl (appropriate isotype controls and single staining were also done, not shown). The nuclei were counter stained with (C and I) DAPI at 1μg/ml. Overlapping signals are shown in D and J. Cells were mounted using anti-fade media. Confocal microscopy was performed using an Olympus microscope. All images corrected for background–thresholds determined by DAPI alone, MsIg-FITC alone, and MsIg-PE alone. Co-localization analysis was run using an ImageJ add-in, red and green pixels that co-localize are shown in white (E, CD13-MMP14; K, CD13-CD90) and the scatter plots of co-localization are shown in F and L respectively. Representative of n = 6 (TIF) [file pone.0162008.s002.tif]

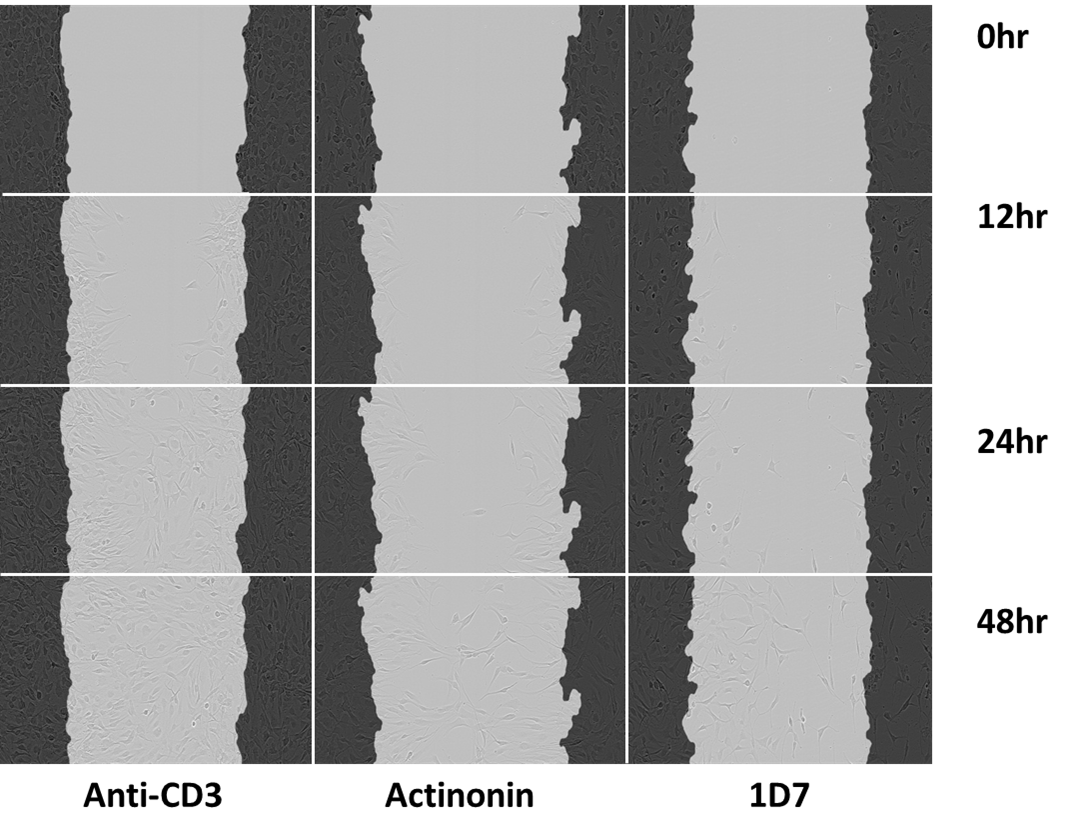

Supplement: S3 Fig — FLS were seeded on 96-well plates overnight. An Essen Incucyte system was used for scratch wounds and migration measurements. Migration was measured in a scratch wound assay using relative wound density. Representative of n≥4. (TIF) [file pone.0162008.s003.tif]
